# Atraumatic scapular body fractures after reverse total shoulder arthroplasty: a case series

**DOI:** 10.1016/j.xrrt.2024.01.007

**Published:** 2024-02-16

**Authors:** Purav S. Brahmbhatt, Randall J. Otto

**Affiliations:** Department of Orthopedic Surgery, Saint Louis University School of Medicine, St. Louis, MO, USA

**Keywords:** Reverse total shoulder arthroplasty, Periprosthetic fracture, Scapular fracture, Notching, Scapular body fracture, Acromial stress fracture

Reverse total shoulder arthroplasty (RSA) has become a well-accepted and increasingly utilized procedure for the treatment of complex shoulder pathology in recent years.^10^^,^^11^^,^^24^^,^^25^ With the increased volume of procedures performed,^8^ the expanded indications for surgery in diverse age groups,^5^^,^^7^^,^^9^^,^^13^ and the expected increased future demand for the procedure,^21^ it is necessary to study complications of the procedure in detail.^1^^,^^3^^,^^6^^,^^12^ Much has been studied about the reverse total shoulder prosthesis design, technical challenges, and outcomes in both the short term and long term.^24^, ^25^, ^26^ Large multicenter trials have previously described a number of complications of reverse total shoulder arthroplasty, including acromial stress fractures, scapular spine fractures, scapular notching, implant loosening, neurologic injury, and infection.^24^^,^^25^ The goal of this article is to report on patients with atraumatic scapular body fractures with and without loosening of the glenoid component. These fractures are not due to trauma, such as a fall. Further, they are not related directed to an iatrogenic injury during surgery. Additionally, these fractures are a unique pathology differentiated from the described scapular spine and acromial stress fractures, which are due to either deltoid over-tensioning or abutment of the greater tuberosity on the underside of the acromion, respectively.^12^^,^^15^^,^^25^^,^^27^ In this article, we present a case series of atraumatic fractures of the scapular body in patients who have undergone RSA and describe patient characteristics, implants, management strategies, and outcomes.

The authors received written consent from the patients for publication of this manuscript.

## Case 1

**Figure 1:**
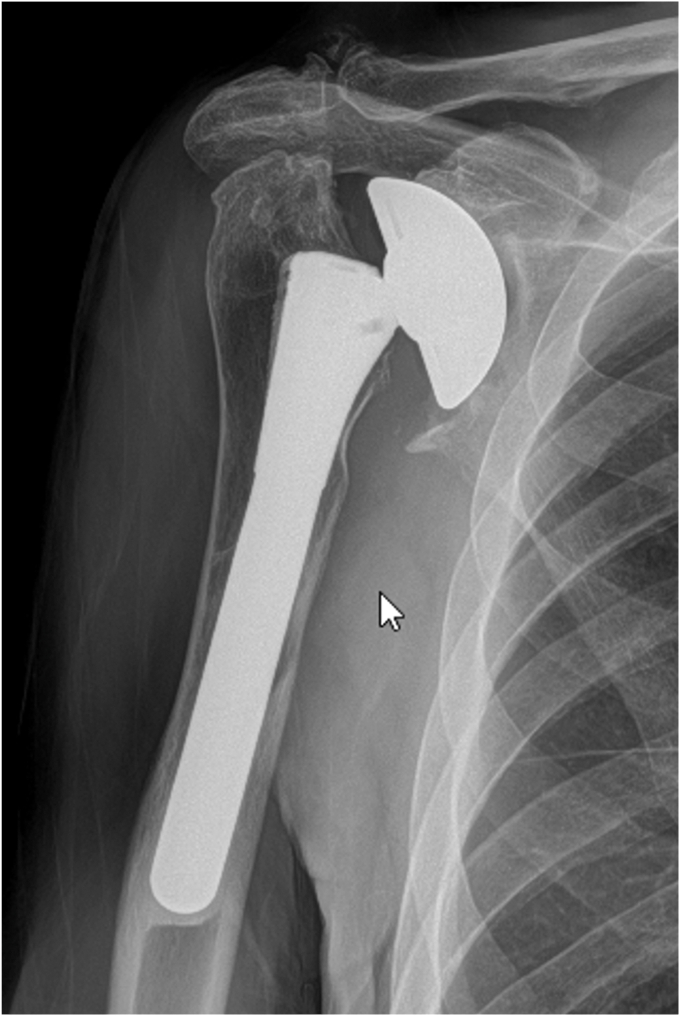
X-ray Grashey view demonstrating the preoperative right shoulder bipolar hemiarthroplasty with prominent glenoid bone loss.

**Figure 2:**
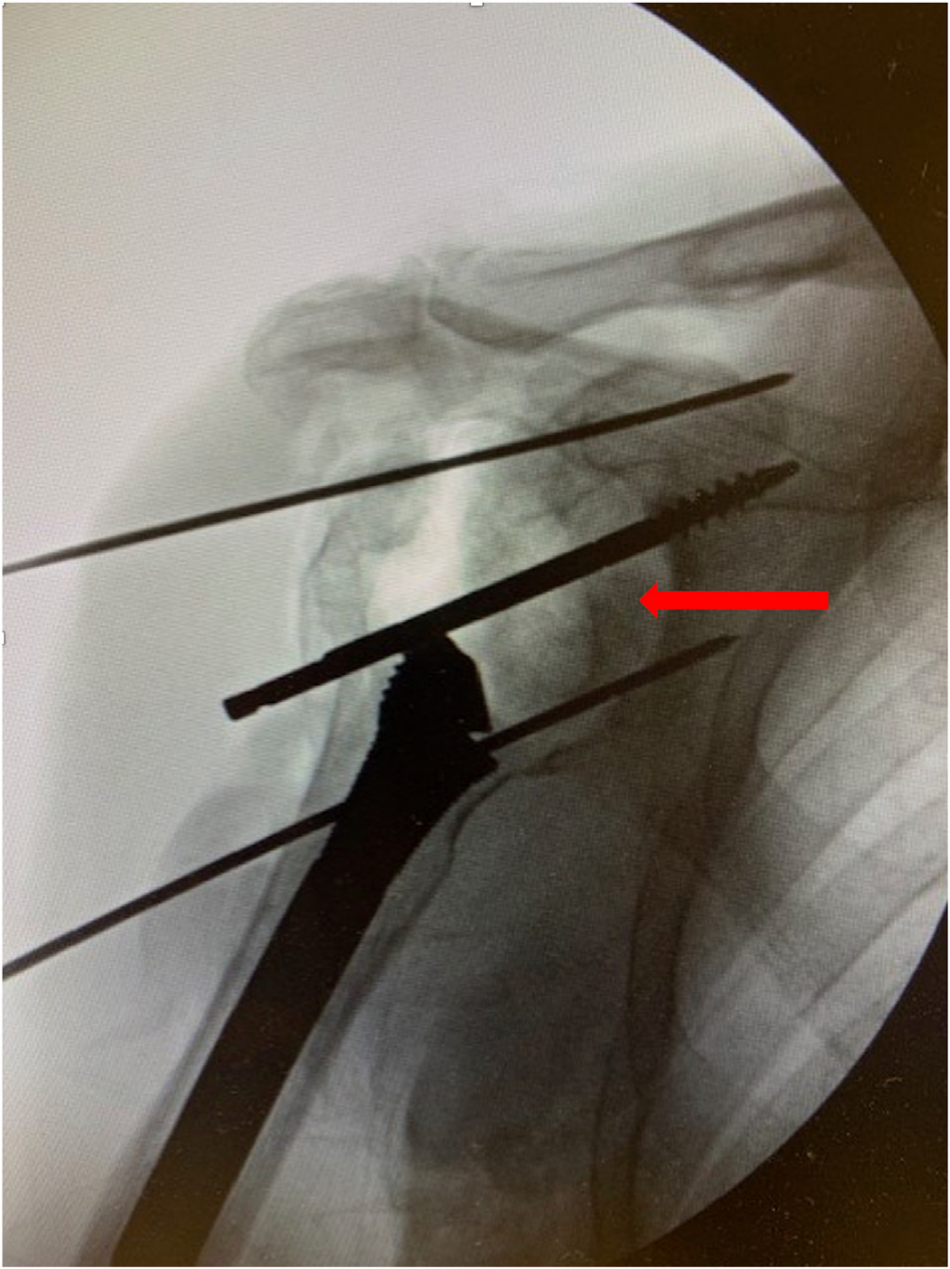
Intraoperative fluoroscopic image showing femoral head bone graft application into the glenoid bone defect with Kirschner wires and a tap. *Red arrow* points to the bone graft.

**Figure 3:**
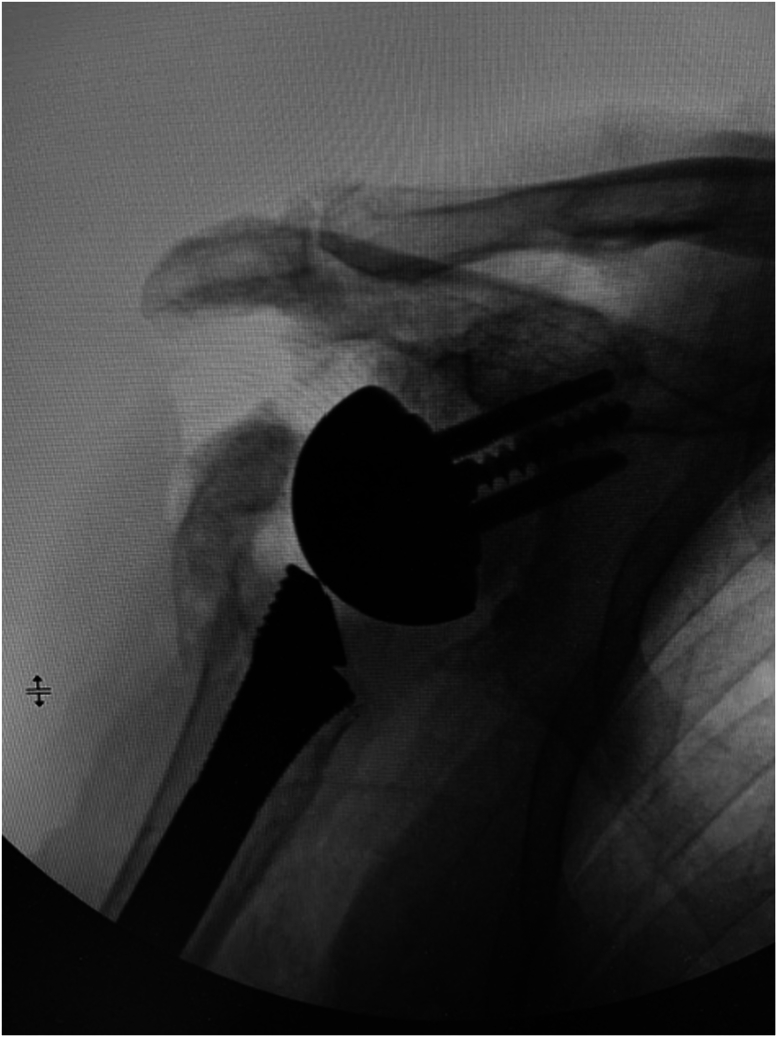
Intraoperative fluoroscopic image showing interval placement of a glenoid base plate, locking screws, and a hooded glenosphere.

**Figure 4:**
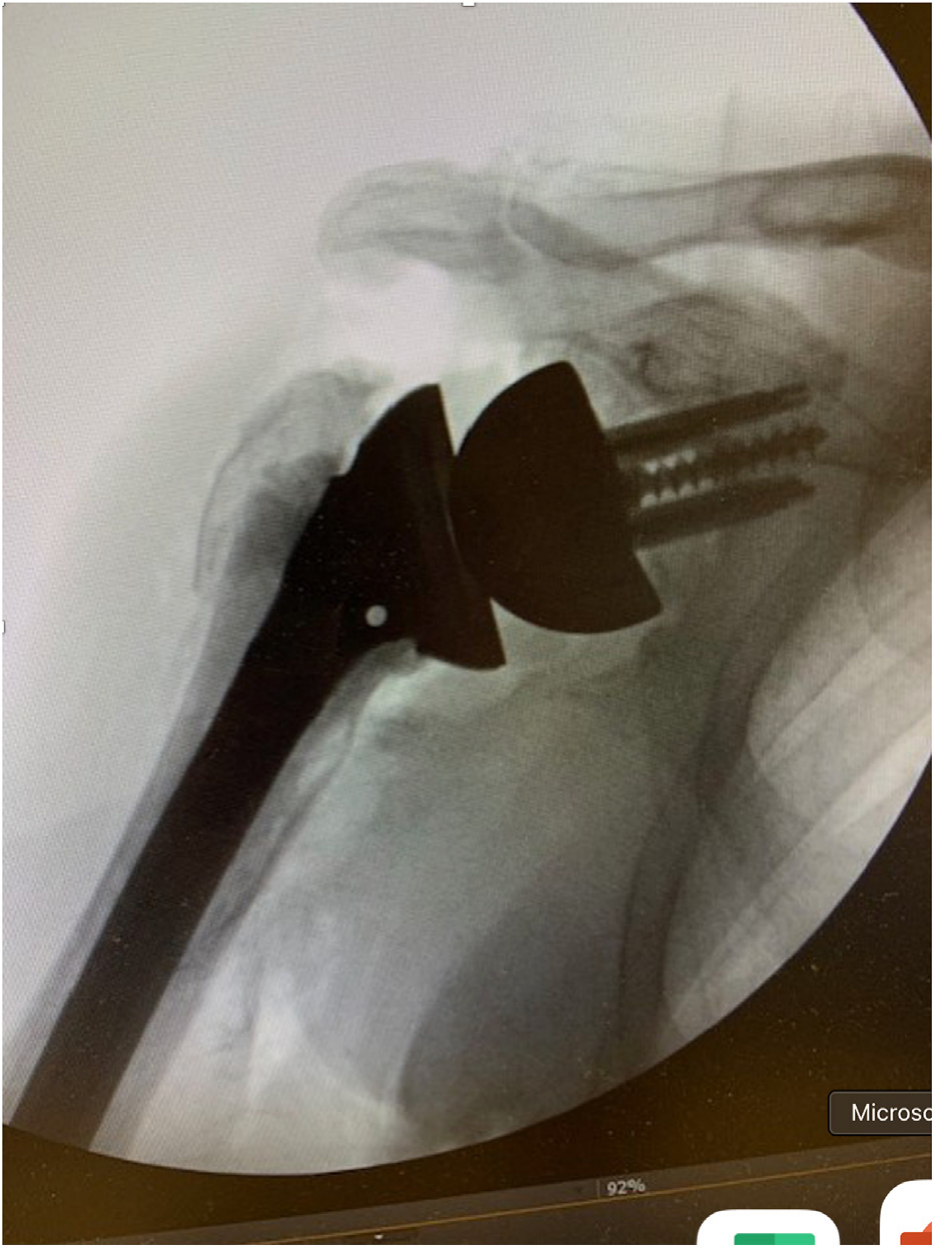
Final intraoperative fluoroscopic image showing an adequately positioned glenoid implant with underlying bone graft to correct the preoperative glenoid defect.

A 73-year-old man presented with a history of a right bipolar shoulder hemiarthroplasty and left RSA who had been developing worsening pain and paresthesia in his right arm. He had originally undergone a right bipolar shoulder hemiarthroplasty after sustaining a direct, traumatic shoulder injury, and his left reverse total shoulder arthroplasty was due to rotator cuff arthropathy. Because of worsening symptoms in his right shoulder that were not relieved with conservative measures, he was looking for treatment options. He underwent a computed tomography (CT) scan that demonstrated significant posterosuperior glenoid erosion and an acromioclavicular joint cyst. An electromyographic study noted that he had developed right median neuropathy consistent with carpal tunnel syndrome. He elected to revise his right shoulder hemiarthroplasty to a RSA with glenoid bone grafting, excise his acromioclavicular cyst, and release his carpal tunnel. The preoperative radiograph of his right shoulder hemiarthroplasty is presented (Fig. 1). For the reverse total shoulder, a standard deltopectoral approach was performed. The previous bipolar hemiarthroplasty implant was removed through standard techniques. Intraoperatively, prominent metallosis of the joint capsule was noted. The glenoid bone surface was prepared to receive bone grafting. The implant chosen for the revision was the Enovis Altivate Reverse Shoulder Prosthesis (Enovis, Wilmington, DE, USA) because of a center screw on the baseplate making it amenable to bone grafting techniques with high success.^14^ The alternative scapular spine line technique^14^ was used to ensure the center fixation of the baseplate would be in native bone. Once a tap was placed, a femoral head allograft was fashioned and placed into position to allow for compression of the graft between the baseplate and native bone (Fig. 2). Locking screws were placed through the graft and into native scapular bone. A hooded glenosphere was used in order to place the hood over the graft to allow for further compression of the graft against the native glenoid bone (Fig. 3). No intraoperative complications were noted, including fracture either in the humerus, clavicle, or scapula. The final intraoperative fluoroscopic film of the right shoulder demonstrated adequate positioning of the glenosphere and incorporation of the bone graft (Fig. 4).

**Figure 5:**
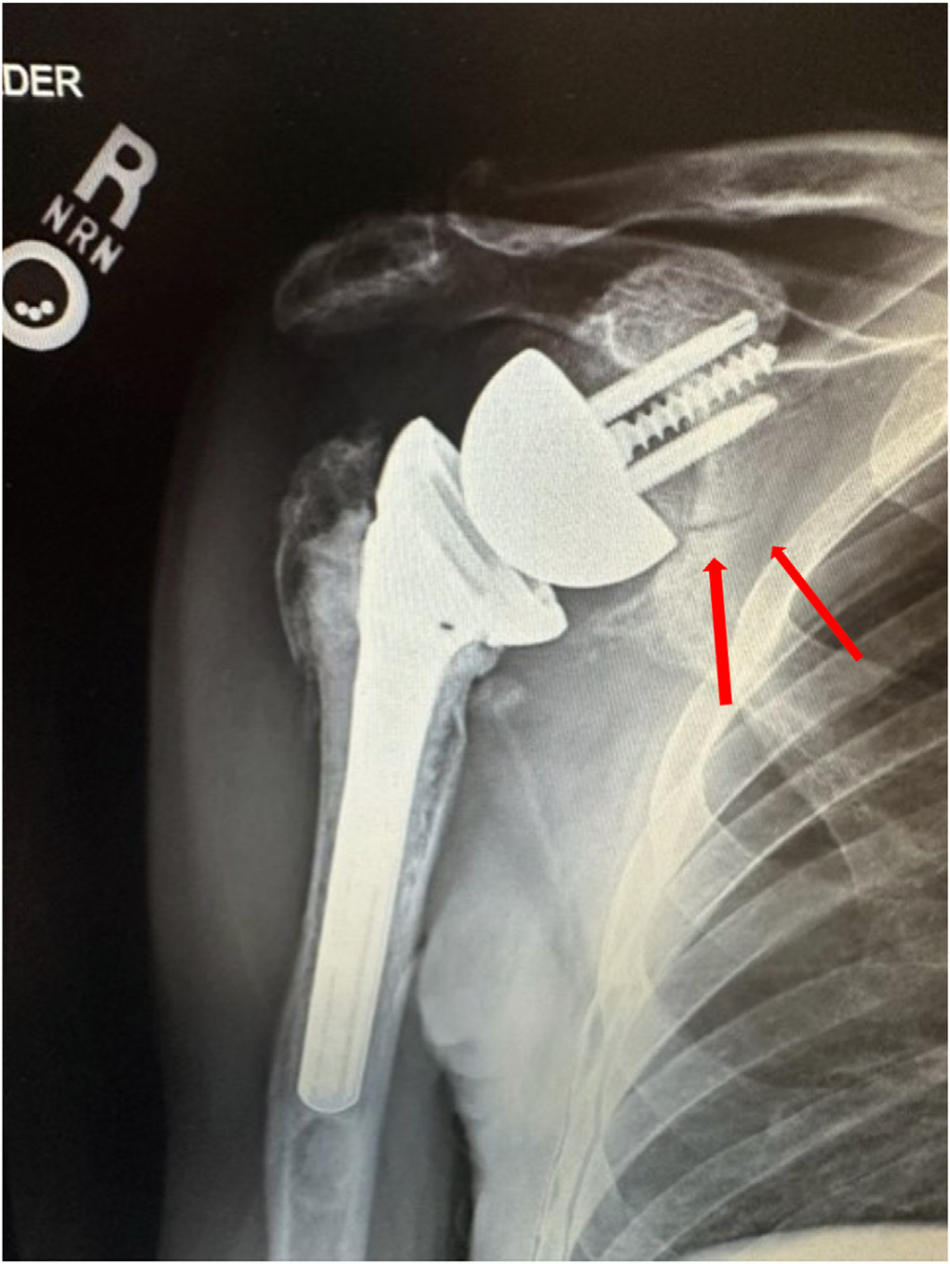
X-ray Grashey view demonstrating an incomplete fracture of the scapular body.

**Figure 6:**
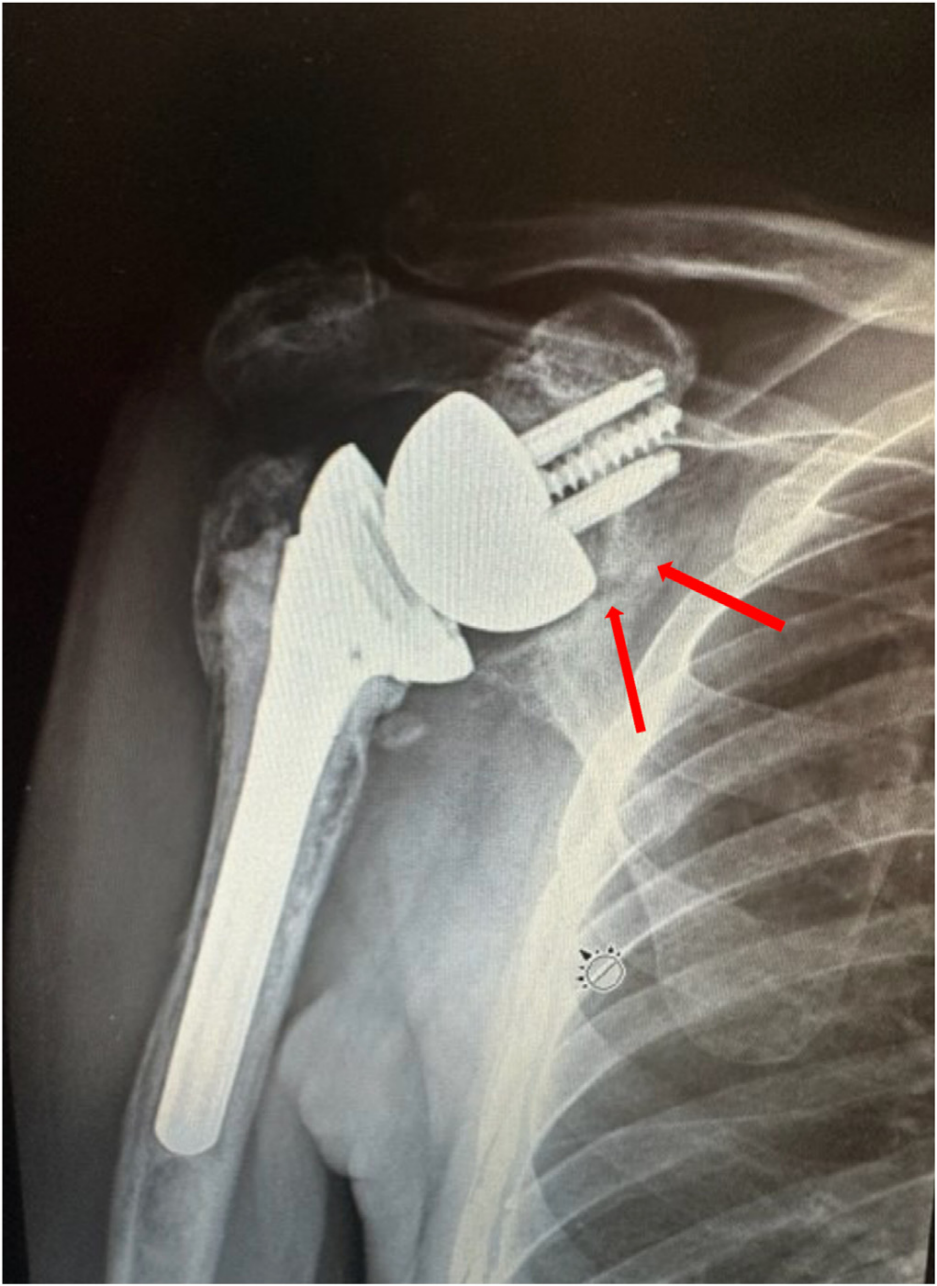
X-ray Grashey view demonstrating healing of the fracture at the scapular body.

**Figure 7:**
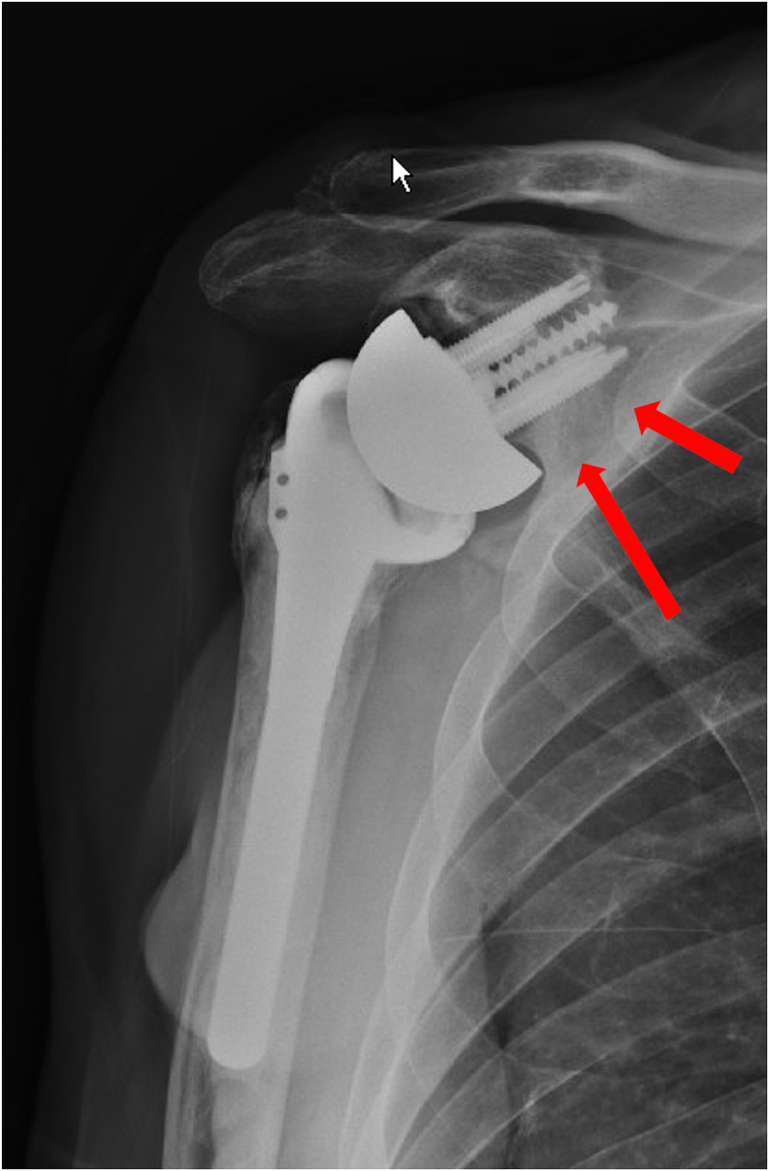
X-ray Grashey view demonstrating a healed fracture at the scapular body.

At the 6-week follow-up, the radiographs of the shoulder showed stable position and alignment of the implant. Supine active assist forward elevation cane exercises were initiated with a home exercise program, and he was allowed to use his arm for simple daily activities of eating, dressing, and bathing but not lifting more than 1 pound. He presented 2 weeks later with increased shoulder pain. Radiographs at that time revealed that he had developed an incomplete fracture of the scapular body at the area of contact of the interior glenosphere to the native scapula (Fig. 5). This was below the baseplate and did not involve the loosening of the baseplate. It was likely related to a stress response of the glenosphere on the native bone during early activities. He was placed back into a sling for 6 weeks with pendulums, elbow, wrist, and hand motion only. His pain had improved 6 weeks later, and radiographs revealed his fracture was healing. Range of motion was initiated again, and he was allowed to use the arm for simple daily activities. The fracture healed 3 months after occurrence (Fig. 6). He then progressed to strengthening exercises and increased activities as tolerated. Preoperatively, forward elevation was 95 degrees, external rotation was 15 degrees, and internal rotation was S1. His preoperative Simple Shoulder Test (SST) was 0 and his American Shoulder and Elbow Surgeons (ASES) score was 31.67. Postoperatively, at his 3-year follow-up, his radiographs showed complete fracture healing (Fig. 7), and his clinical exam demonstrated forward elevation of 120 degrees, external rotation of 70 degrees, and internal rotation of L5. His SST was 11, and his ASES was 93. He had no residual pain and was performing activities as tolerated.

## Case 2

**Figure 8:**
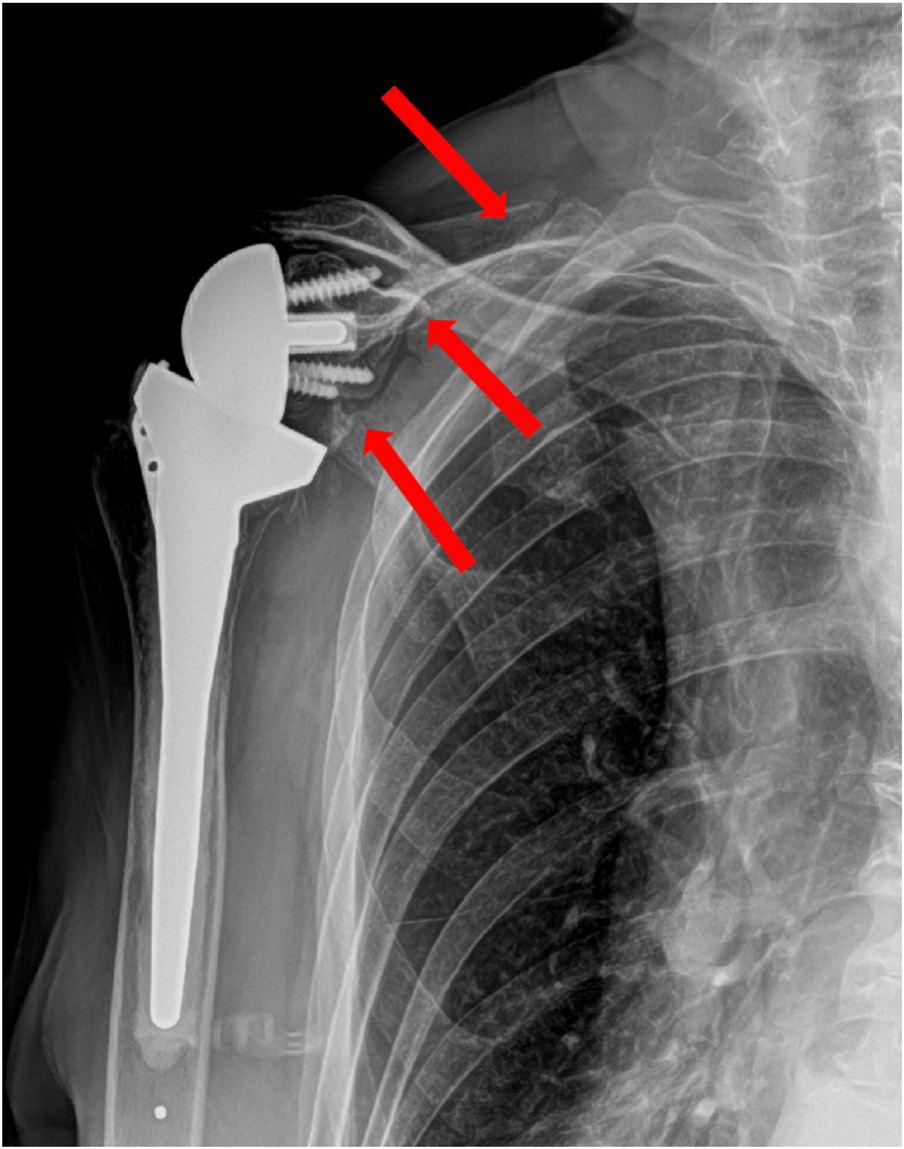
X-ray Grashey view demonstrating a scapular fracture from the inferior scapular neck and exiting the medial scapular spine.

**Figure 9:**
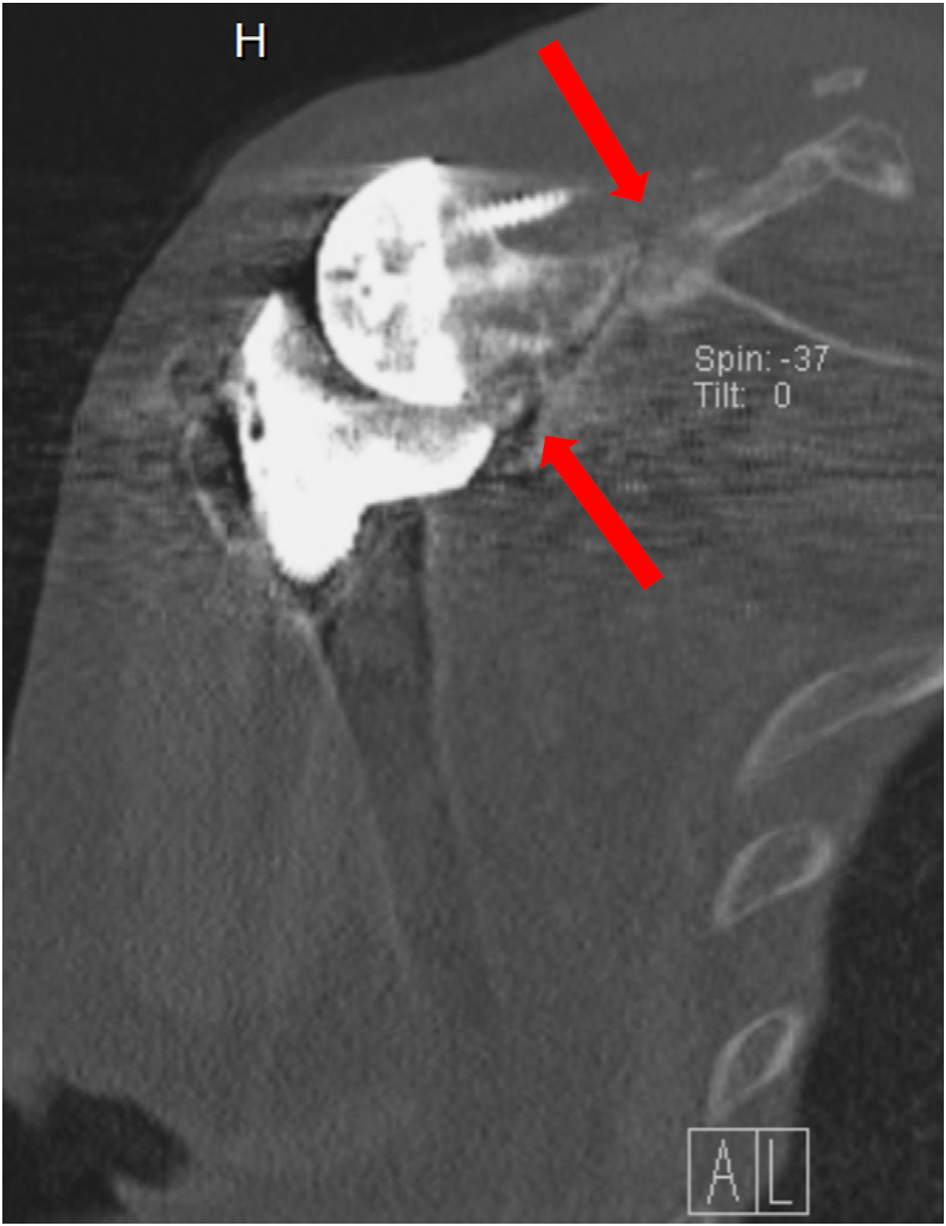
CT scan demonstrating a fracture inferior to the glenoid component. *CT*, computed tomography.

A 73-year-old woman had a history of bilateral reverse total shoulder arthroplasties by an outside physician, presented in June 2022 with worsening pain and loss of function in her right shoulder. She underwent a left RSA in September 2019 for a failed open rotator cuff repair, and she underwent a right RSA in March 2015 by the same surgery for a rotator cuff arthropathy. She noted no recent history of a direct shoulder trauma or injury. She presented to an urgent care and radiographs were concerning for a failed baseplate. She was referred to our clinic for evaluation and revision surgery. Radiographs at that time showed a fracture beginning at the inferior scapular neck, extending across the scapular body, and exiting the medial scapular spine (Fig. 8). A CT scan confirmed a fracture inferior to the glenoid component with a “floating glenoid” similar to a previously reported case (Fig. 9).^4^ The glenoid component was found to be stable and well fixed. It is postulated that the fracture occurred late after chronic impingement and subsequent notching from the humeral component. The patient’s prefracture function was limited to forward elevation of 90 degrees, external rotation of 40 degrees, and internal rotation to L4.

**Figure 10:**
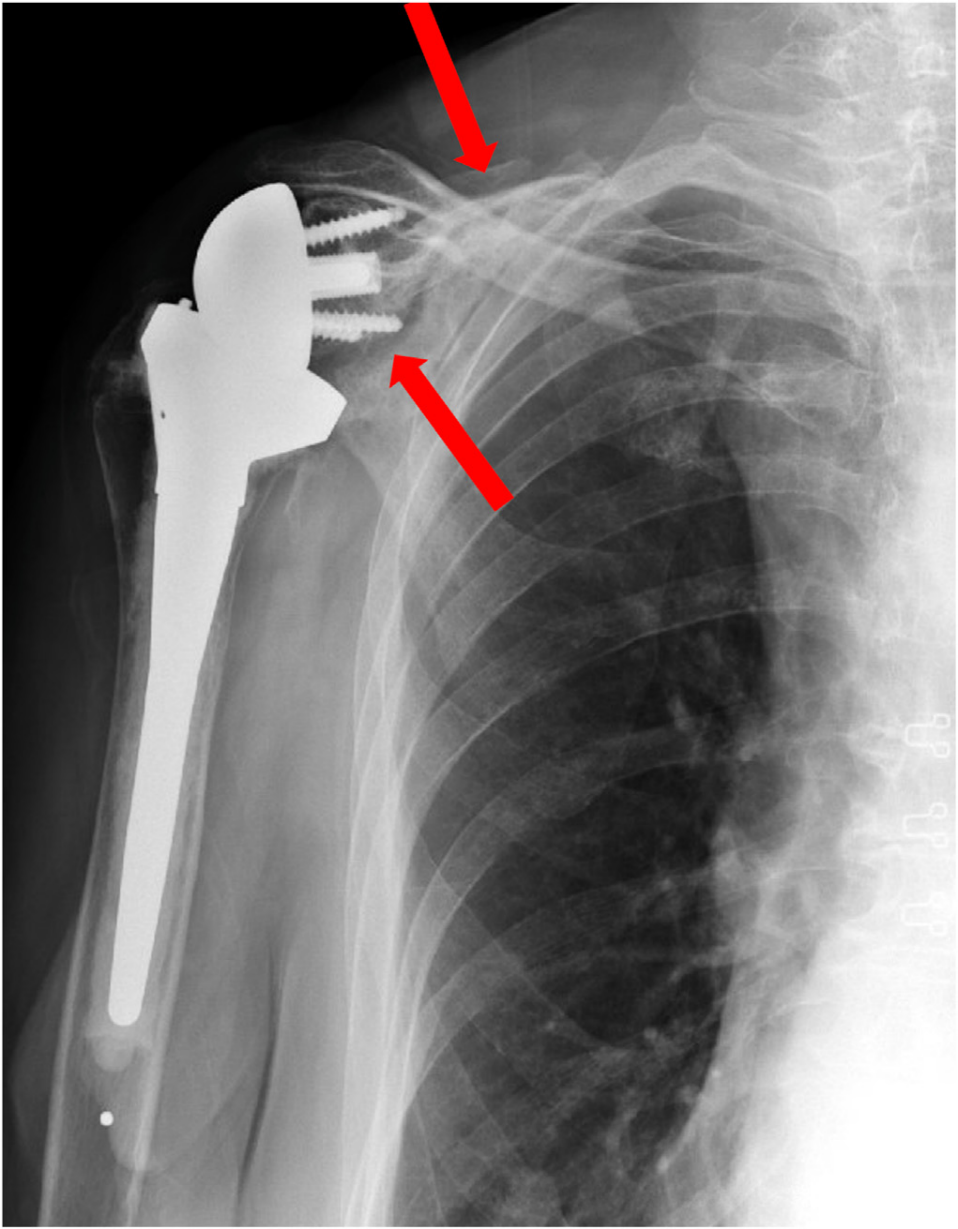
X-ray Grashey view demonstrating healing of the scapular body fracture.

**Figure 11:**
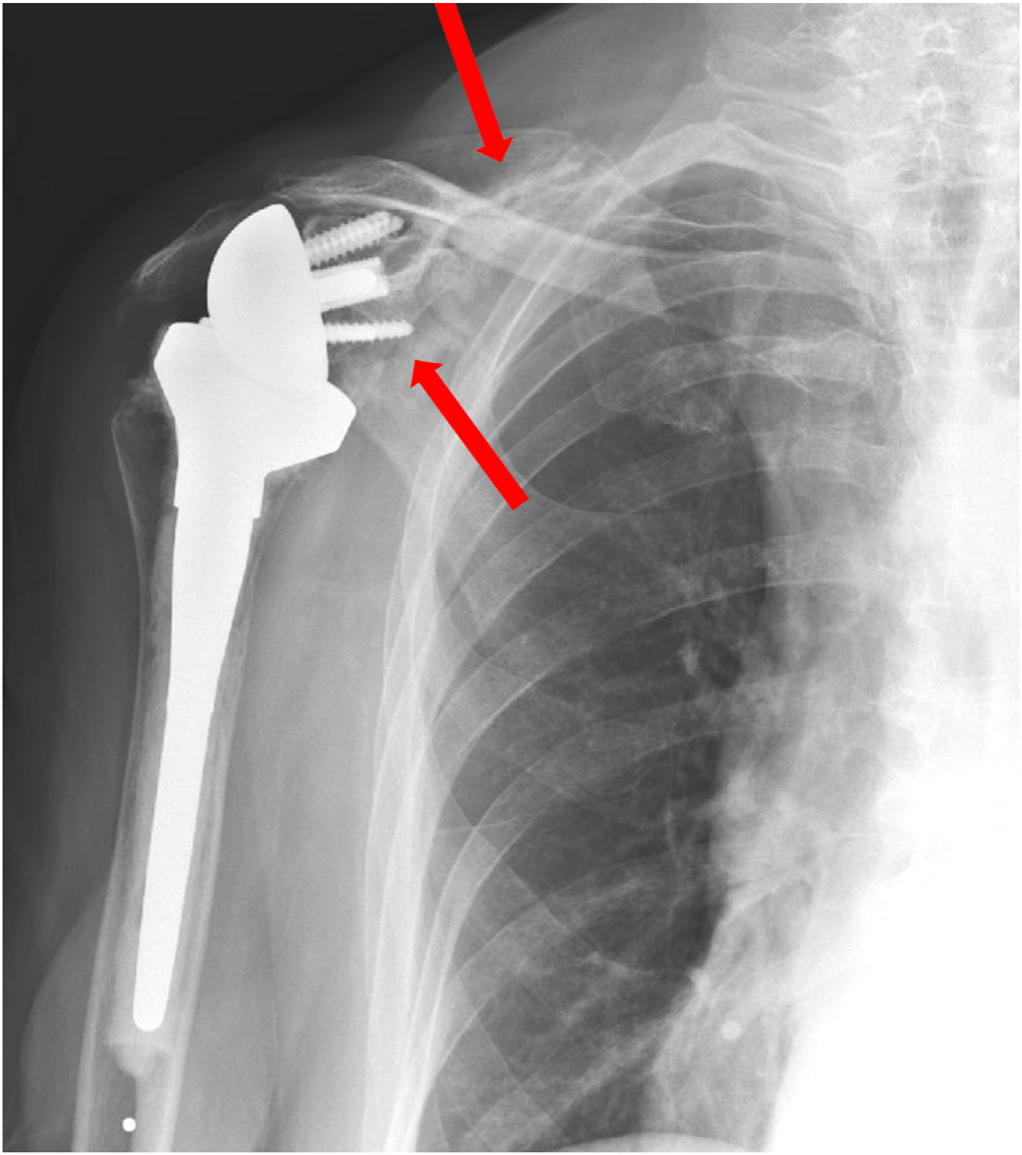
X-ray Grashey view demonstrating continued healing of the scapular body fracture.

Because the implant was stable, the fracture was minimally displaced, and her pre-fracture function was limited, we elected to treat her conservatively with a course of sling immobilization. At her appointment 5 weeks after the fracture, her pain was improving and her radiographs revealed healing of the fracture (Fig. 10). We initiated home supine active assist forward elevation cane exercises and allowed her to use her arm for simple daily activities of eating, dressing, and bathing. At 3 months from fracture, her pain was resolved and the fracture had healed on radiographs (Fig. 11). At 5.5 months from her fracture, her function had returned to her baseline of forward elevation of 90 degrees, external rotation of 40 degrees, and internal rotation to L4. She had no pain and returned to activities as tolerated.

## Case 3

**Figure 12:**
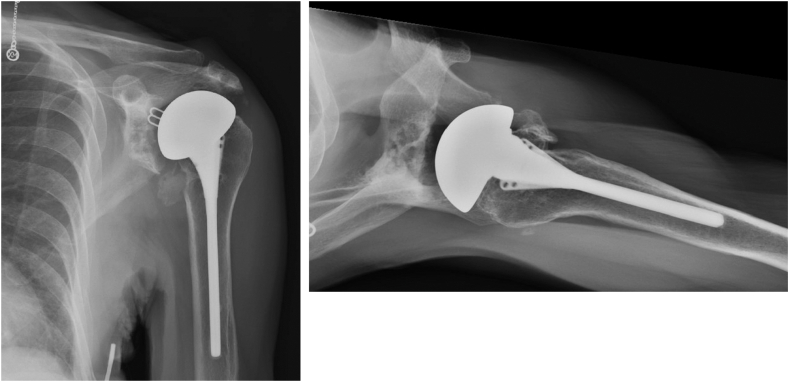
X-ray Grashey and axillary lateral view demonstrating no obvious fracture line in the shoulder.

**Figure 13:**
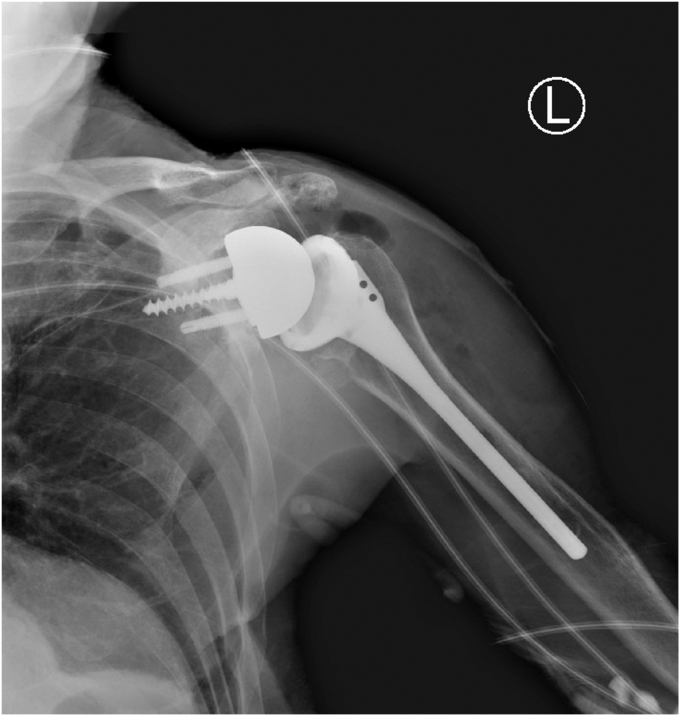
X-ray showing a postoperative image of a reverse total shoulder arthroplasty.

**Figure 14:**
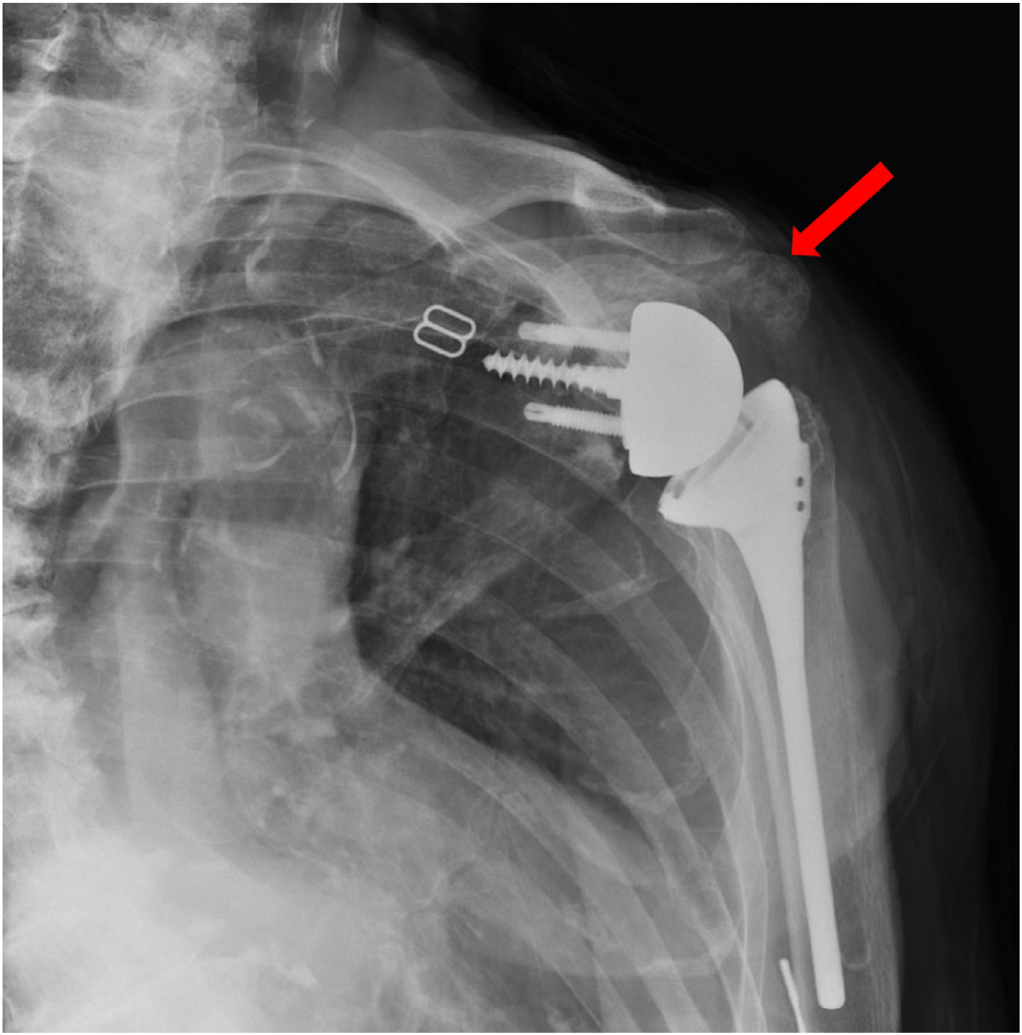
X-ray Grashey view demonstrating an acromial fracture with inferior tilt.

**Figure 15:**
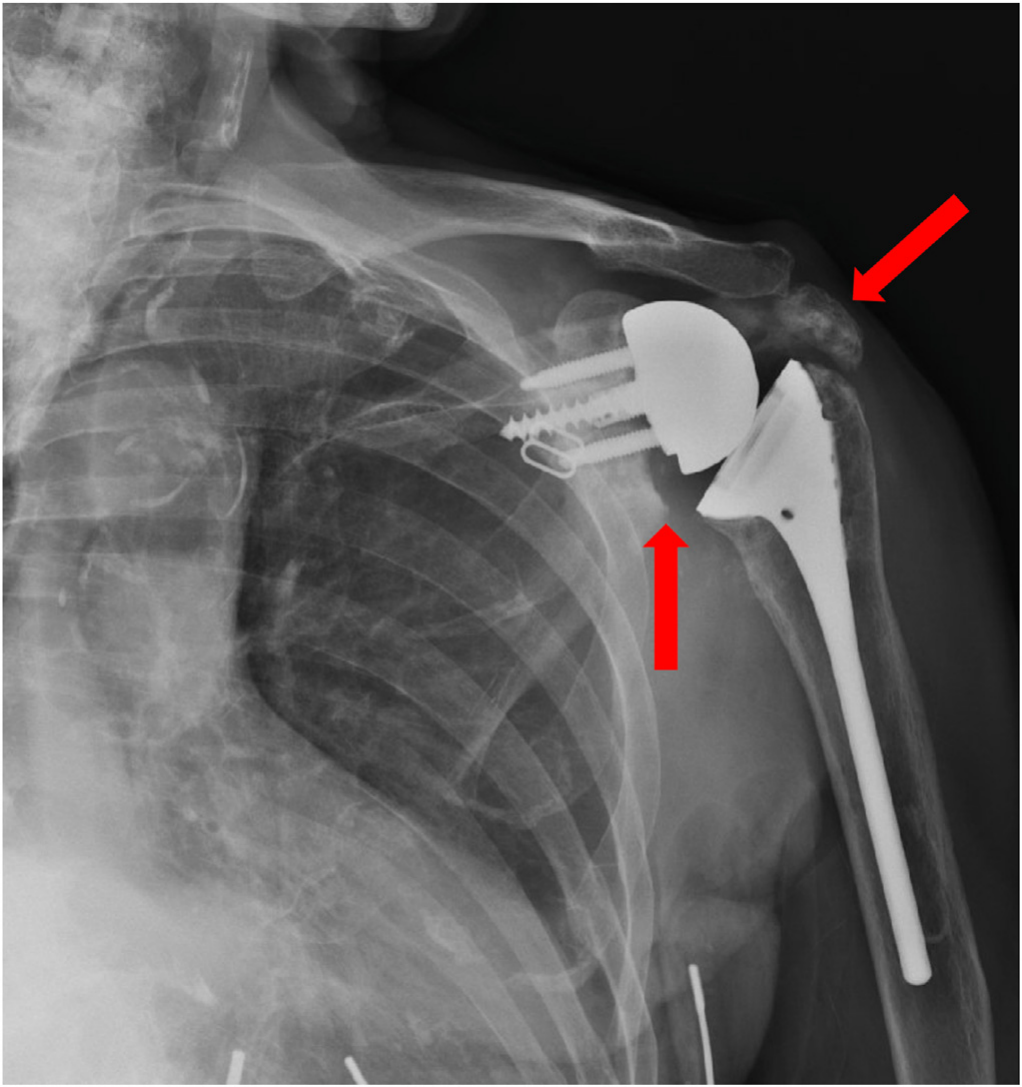
X-ray Grashey view demonstrating a change in glenoid component positioning relative to prior imaging in Fig. 10.

**Figure 16:**
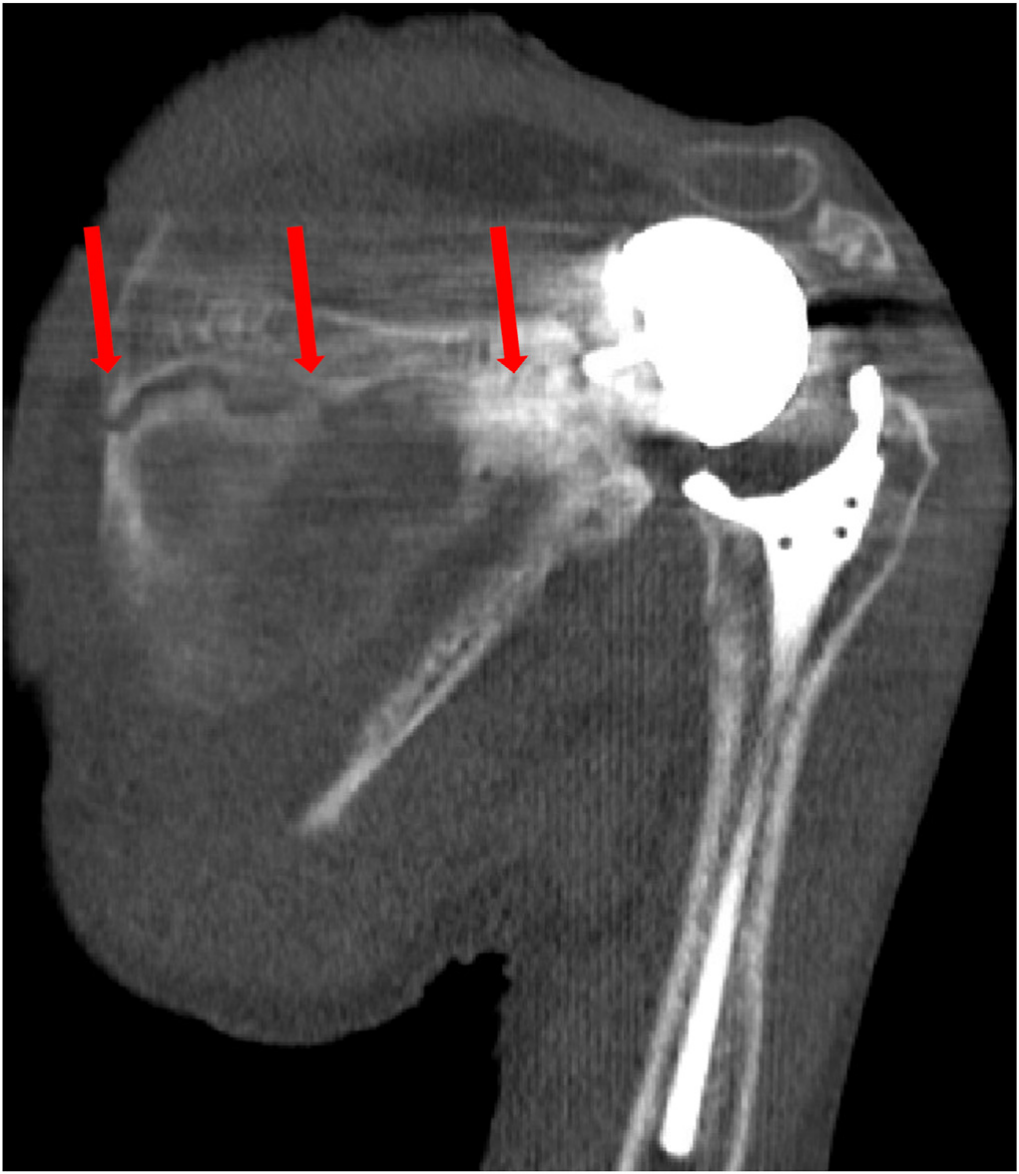
CT scan showing peri-implant scapular fracture with signs of glenoid loosening. *CT*, computed tomography.

**Figure 17:**
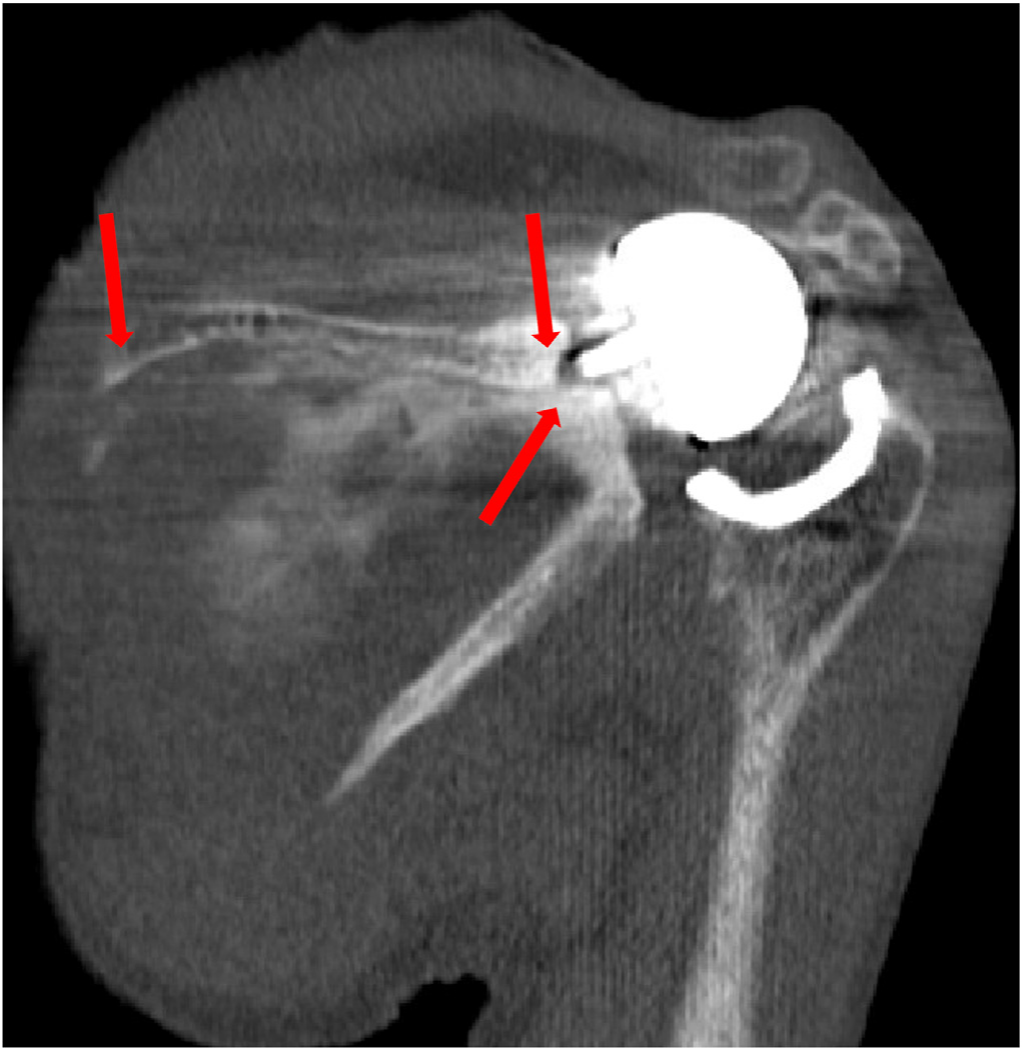
CT scan showing peri-implant scapular fracture with signs of glenoid loosening. *CT*, computed tomography.

**Figure 18:**
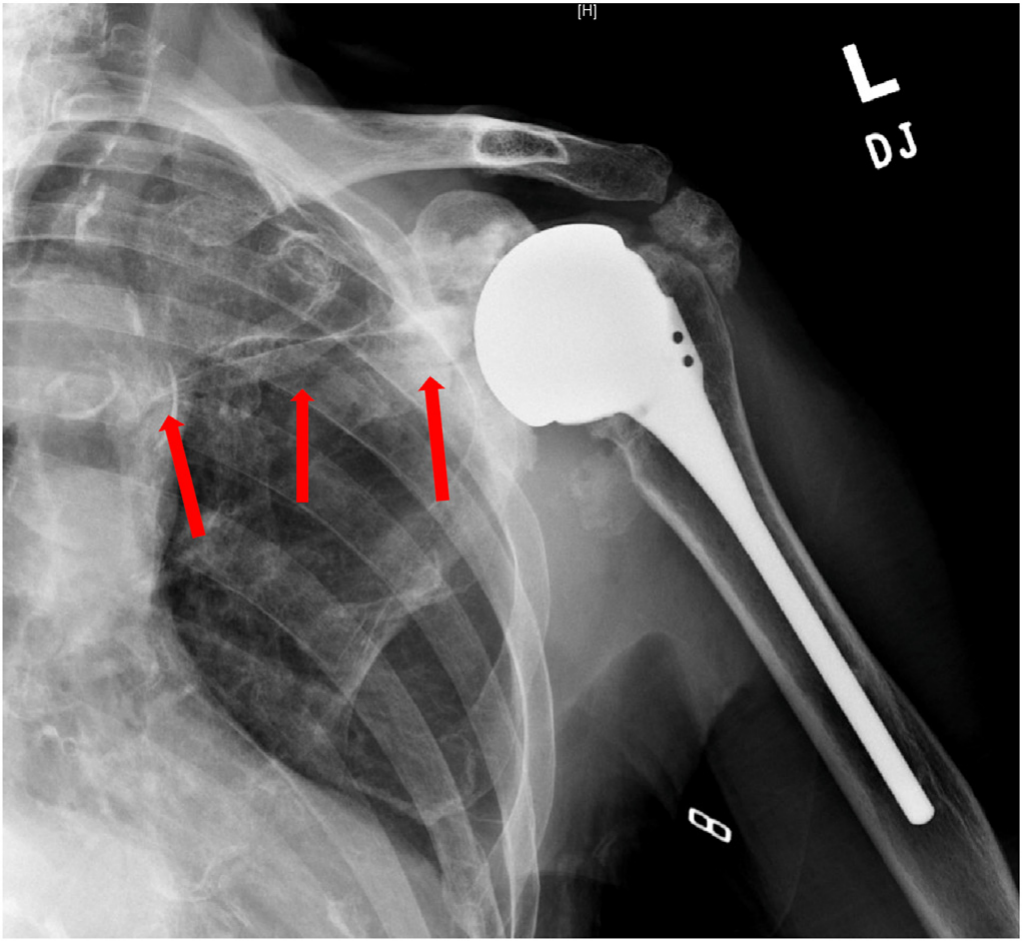
X-ray Grashey view demonstrating stable position of implant with signs of fracture healing.

An 80-year-old female with a history of bilateral anatomic shoulder arthroplasties presented for evaluation for approximately one month of pain in the left shoulder after reportedly lifting trash to throw in a bin. She was roughly seven years status post her left anatomic total shoulder at the time. She noted that her left shoulder was in pain at rest and with motion. Radiographs at the time demonstrated no fracture (Fig. 12), and a later CT arthrogram showed a left-sided rotator cuff tear with signs of a loose glenoid component. She then elected for surgical management, and her anatomic total shoulder arthroplasty was converted to a reverse total shoulder arthroplasty (Fig. 13). She was doing well with her left reverse total shoulder postoperatively. At the three-month follow-up visit, however, she noted continued shoulder pain in the deltoid region. Radiographs demonstrated an acromial fracture with inferior tilt (Fig. 14). She was sent for formal physical therapy for her left shoulder to work on modalities, stretching, and deltoid strengthening. At the four-and-a-half-month mark from surgery, she did not have improvement. Her subsequent radiographs demonstrated a new change in her glenoid component position concerning for loosening (Fig. 15). An aspiration of her joint was performed, and it was negative for infection with a Synovasure (Zimmer Biomet, Warsaw, IN, USA) analysis. She also underwent a CT scan that showed a peri-implant scapular fracture with signs of glenoid loosening (Figure 16, Figure 17 and Figure 16, Figure 17). We discussed both operative and nonoperative options, and she elected for surgery. We then converted her left reverse total shoulder arthroplasty to a revision hemiarthroplasty with the removal of the glenoid component and glenoid bone grafting. Intraoperatively, gross instability of the glenosphere was noted. A stellate fracture of the glenoid extending to the anteroinferior glenoid, lateral scapular column, and superior scapular spine with a free-floating coracoid was also noted. The soft tissue around the glenoid was débrided until bleeding edges were noted, and a hemi-adaptor was placed onto the humeral stem. The glenoid cavity was packed with cancellous bone allograft. Intraoperative cultures were negative for infection. The patient did well postoperatively, and her pain was significantly reduced. Her radiographs showed a stable position of the implants with signs of healing at the fracture (Fig. 18). At the most recent follow-up at 6 months, the patient was able to perform forward elevation to 90 degrees, abduction to 70 degrees, external rotation to 60 degrees, and internal rotation to L5. Her functional outcome scores at that time revealed a SST of 7, an ASES score of 73, and a Single Assessment Numeric Evaluation of 50.

## Discussion

As RSA is becoming more popular for the treatment of severe shoulder pathology, a number of complications associated with RSA have been described with mid- to long-term follow-up.^6^^,^^16^^,^^24^^,^^25^^,^^27^ Complications include those common to total joint arthroplasty such as infection, instability, mechanical failure, and aseptic loosening. Furthermore, a number of complications that are unique to reverse shoulder arthroplasty such as scapular notching, acromial stress fractures, and scapular spine fractures have also been described.^3^^,^^6^^,^^16^^,^^24^^,^^25^

With continued research and innovation in RSA, the literature shows the rate of many described complications has improved compared to the rates of complications in the original Grammont-style prosthesis.^23^, ^24^, ^25^^,^^28^ Indeed, the rate of scapular notching, scapular spine fractures, and acromial stress fractures has decreased in recent years with modifications in surgical technique and implant design. ^17^^,^^18^^,^^22^^,^^25^ Much of this can be attributed to a better understanding about the implant center of rotation and progress in understanding deltoid tensioning and implant positioning.^22^

While the complications above may be better identified today, the increasing prevalence of RSA with long-term implant durability is now presenting with new, rare complications. One such issue is the development of an atraumatic, peri-implant scapular body fracture that involves the glenoid neck. A case report by Bell et al described such a periprosthetic scapular body fracture and presented a management strategy for this issue.^4^ The case presented a patient with a history of a reverse total shoulder arthroplasty that developed an unstable periprosthetic glenoid neck fracture with baseplate loosening that required a revision to a hemiarthroplasty.^4^

The fractures described in our series are similar to the above and differentiated from acromial stress fractures and scapular spine fractures both by their orientation and presumably also by their pathophysiology. While there is debate about the mechanism of these complications, much of the current literature has hypothesized that deltoid over-tensioning in soft, osteoporotic bone and screw placement all play a role.^2^^,^^12^^,^^15^^,^^17^^,^^19^^,^^20^

In our study, we identified three patients with this similar pattern. One patient had undergone a primary RSA for cuff tear arthropathy while the other two were status post a revision RSA for a failed shoulder arthroplasty. One patient developed an incomplete fracture inferior to the baseplate. Another developed a fracture inferior to the baseplate extending medially and exiting the scapular spine. The final patient developed a stellate glenoid vault fracture extending through the medial border of the scapula.

In our management of these fractures, we immediately immobilized our patients in slings. We obtained advanced imaging with CT scans to further evaluate the extent of injury and delineate implant involvement with the fracture. We found that two patients had stable glenoid implants and were treated with sling immobilization definitively. One, however, had a loose glenoid baseplate and was ultimately revised to a hemiarthroplasty. All three patients went on to fracture healing, resolution of pain, and return to baseline functional range of motion.

## Conclusion

Although we do not know the exact cause of the fractures, we can postulate the cause in these cases. One fracture could be a result of mechanical stress due to load-sharing of the glenosphere around the baseplate, as seen in one patient with a hooded glenosphere which contacted the inferior glenoid at the location of the incomplete fracture. Another cause could be late-term scapular notching on the glenoid, as seen in a patient with a fracture inferior to the baseplate along the neck and extending into the medial scapular spine. Finally, baseplate screw fixation in osteoporotic bone with glenoid bone loss from prior failed arthroplasty could also contribute to these fractures, which is different from a failed baseplate after revision for a failed anatomic shoulder arthroplasty.

Further biomechanical studies are warranted, and more reports of these fractures are necessary for larger group analysis. We present this series to highlight along with Bell et al^4^ that further research into this fracture pattern and its pathogenesis can help guide improved future implant design, patient counseling, surgical technique, and management strategies.

## Disclaimers:

Funding: No funding was disclosed by the authors.

Conflicts of interest: Randall Otto, MD, is a consultant and designer for Enovis and receives consulting fees. He is also a consultant for Restor3d and receives consulting fees. He serves on a committee for American Shoulder and Elbow Surgeons. The other author, his immediate family, and any research foundation with which he is affiliated have not received any financial payments or other benefits from any commercial entity related to the subject of this article.

Patient consent: Obtained.
